# Nutritional disorders in the proposed 11th revision of the International Classification of Diseases: feedback from a survey of stakeholders

**DOI:** 10.1017/S1368980016001427

**Published:** 2016-06-13

**Authors:** Mercedes de Onis, Julia Zeitlhuber, Cecilia Martínez-Costa

**Affiliations:** 1 Department of Nutrition for Health and Development, World Health Organization, 20 Avenue Appia, 1211 Geneva 27, Switzerland; 2 Department of Nutritional Science, University of Vienna, Vienna, Austria; 3 Department of Pediatrics, University of Valencia, Valencia, Spain

**Keywords:** ICD-11, Classification, Nutritional disorders, WHO

## Abstract

**Objective:**

To receive stakeholders’ feedback on the new structure of the Nutritional Disorders section of the International Classification of Diseases, 11th Revision (ICD-11).

**Design:**

A twenty-five-item survey questionnaire on the ICD-11 Nutritional Disorders section was developed and sent out via email. The international online survey investigated participants’ current use of the ICD and their opinion of the new structure being proposed for ICD-11. The LimeSurvey^®^ software was used to conduct the survey. Summary statistical analyses were performed using the survey tool.

**Setting:**

Worldwide.

**Subjects:**

Individuals subscribed to the mailing list of the WHO Department of Nutrition for Health and Development.

**Results:**

Seventy-two participants currently using the ICD, mainly nutritionists, public health professionals and medical doctors, completed the questionnaire (response rate 16 %). Most participants (*n* 69) reported the proposed new structure will be a useful improvement over ICD-10 and 78 % (*n* 56) considered that all nutritional disorders encountered in their work were represented. Overall, participants expressed satisfaction with the comprehensiveness, clarity and life cycle approach. Areas identified for improvement before ICD-11 is finalized included adding some missing disorders, more clarity on the transition to new terminology, links to other classifications and actions to address the disorders.

**Conclusions:**

The Nutritional Disorders section being proposed for ICD-11 offers significant improvements compared with ICD-10. The new taxonomy and inclusion of currently missing entities is expected to enhance the classification and health-care professionals’ accurate coding of the full range of nutritional disorders throughout the life cycle.

The International Classification of Diseases (ICD) is the standard diagnostic tool for epidemiology, health management and clinical purposes. This includes the analysis of the general health situation of population groups. Most countries use the ICD to report mortality data, a primary indicator of health status, as well as to monitor the incidence and prevalence of diseases and other health problems, providing a picture of the general health situation of countries and populations.

The ICD is used by physicians, nurses, other providers, researchers, health information managers and coders, health information technology workers, policy makers, insurers and patient organizations to classify diseases and other health problems recorded on many types of health and vital records, including death certificates and health records. In addition to enabling the storage and retrieval of diagnostic information for clinical, epidemiological and quality purposes, these records also provide the basis for the compilation of national mortality and morbidity statistics. Notably, the ICD is used for reimbursement and resource allocation decision making by countries^(^ ^1^ ^)^.

Since its 6th revision in 1948, the WHO has undertaken periodic revisions of the ICD. Clinical modifications of the ICD have been developed and implemented to accommodate country-specific needs for classifying diagnoses in coded health data^(^ ^2^ ^–^ ^6^ ^)^.

It is more than 20 years since the Forty-third World Health Assembly (May 1990) endorsed the tenth ICD revision (ICD-10) and WHO Member States adopted it for clinical use. WHO is currently working on the 11th revision, which the World Health Assembly is expected to approve in May 2018. The rationale for the revision is to reflect progress in the understanding of health and disease, improve its clinical utility and adapt the classification to advances in information technology^(^ ^7^ ^)^. Among the main changes proposed there are many new elements, such as: new chapters (e.g. diseases of the blood and blood-forming organs, disorders of the immune system, conditions related to sexual health, sleep–wake disorders, traditional medicine); restructuring of existing chapters; content model (e.g. all conditions/disorders/diseases will include short and long definitions); new coding scheme; new terminology; and new concepts (e.g. classification hierarchy).

Major improvements are anticipated from a nutrition perspective. The 11th revision will include a Nutritional Disorders (ND) section within the ‘Endocrine, nutritional and metabolic diseases’ chapter (Chapter 6), that has been developed by a Topic Advisory Group for Nutrition. The section will include the full range of nutritional disorders, from undernutrition to overweight and obesity, throughout the life cycle. A detailed description of the various enhancements in structure and content will be reported elsewhere.

To foster public awareness and promotion of ICD-11 and to ensure transparency of the revision process, WHO has established an Internet-based editing platform (http://apps.who.int/classifications/icd11/browse/l-m/en) which enables interested parties to participate in the revision process with proposals for enhancing the content and structure^(^ ^8^ ^)^. A total of 5202 proposals had been received by 31 December 2015 for the twenty-six chapters, of which 154 corresponded to Chapter 6 (‘Endocrine, nutritional and metabolic diseases’). Of these 154, less than one-third corresponded to the ND section. Evaluation studies are also underway to field-test the current ICD-11 draft and assess how it improves the quality of the data.

As part of this process, WHO’s Department of Nutrition for Health and Development undertook a survey to seek stakeholders’ opinions on the new structure of the ND section. The aim was to use feedback to enhance this section of ICD-11 before its finalization.

## Methods

A questionnaire on the ICD ND section was developed centrally and sent to subscribers to the WHO Department of Nutrition for Health and Development’s global mailing list. To ensure clarity throughout the survey, questions were kept short and simple; they included a combination of single-choice, multiple-choice and open-ended questions. The single- and multiple-choice questions had pre-coded answer options. The questionnaire (see online supplementary material) included instructions at the beginning of each section. In addition, to enable participants to review and compare approaches, a link to the online ICD-11 Beta Draft^(^ ^8^ ^)^ was provided for the last section (feedback on the new structure of ICD-11 ND section) together with two documents presenting the current (ICD-10; Table 1) and the proposed new structure (ICD-11) of the ND section (Table 2).

**Table 1:** Structure of the International Classification of Diseases, 10th Revision (ICD-10) Nutritional Disorders

ICD-10 Nutritional Disorders	
Malnutrition	
Kwashiorkor	Dietary calcium deficiency
Nutritional marasmus	Dietary selenium deficiency
Marasmic kwashiorkor	Dietary zinc deficiency
Unspecified severe protein–energy malnutrition	Deficiency of other nutrient elements
Protein–energy malnutrition of moderate and mild degree	Copper deficiency
Moderate protein-energy malnutrition	Iron deficiency
Mild protein–energy malnutrition	Magnesium deficiency
Retarded development following protein–energy malnutrition	Manganese deficiency
Unspecified protein–energy malnutrition	Chromium deficiency
Other nutritional disorders	Molybdenum deficiency
Vitamin A deficiency	Vanadium deficiency
Vitamin A deficiency with conjunctival xerosis	Deficiency of multiple nutrient elements
Vitamin A deficiency with Bitot spots and conjuctival xerosis	Deficiency of other specified nutrient elements
Vitamin A deficiency with corneal xerosis	Deficiency of nutrient element, unspecified
Vitamin A deficiency with corneal ulceration and xerosis	Other nutritional deficiencies
Vitamin A deficiency with keratomalacia	Essential fatty acid (EFA) deficiency
Vitamin A deficiency with night blindness	Imbalance of constituents of food intake
Vitamin A deficiency with xerophthalmic scars of cornea	Other specified nutritional deficiencies
Other ocular manifestations of vitamin A deficiencies	Nutritional deficiency, unspecified
Other manifestations of vitamin A deficiencies	Sequelae of malnutrition and other nutritional deficiencies
Vitamin A deficiency, unspecified	Sequelae of protein–energy malnutrition
Thiamin deficiency	Sequelae of vitamin A deficiency
Beriberi	Sequelae of vitamin C deficiency
Wenicke encephalopathy	Sequelae of rickets
Other manifestations of thiamin deficiency	Sequelae of other nutritional deficiencies
Thiamin deficiency, unspecified	Sequelae of unspecified nutritional deficiency
Niacin deficiency (pellagra)	Obesity and other hyperalimentation
Deficiency of other B group vitamins	Localized adiposity
Riboflavin deficiency	Obesity
Pyridoxine deficiency	Obesity due to excess calories
Deficiency of other specified B group vitamins	Drug-induced obesity
Vitamin B deficiency, unspecified	Extreme obesity with alveolar hypoventilation
Ascorbic acid deficiency	Other obesity
Vitamin D deficiency	Obesity, unspecified
Rickets, active	Other hyperalimentation
Vitamin D deficiency, unspecified	Hypervitaminosis A
Other vitamin deficiencies	Hypercarotenaemia
Deficiency of vitamin E	Megavitamin-B_6_ syndrome
Deficiency of vitamin K	Hypervitaminosis D
Deficiency of other vitamins	Other specified hyperalimentation
Vitamin deficiency, unspecified	Sequelae of hyperalimentation

**Table 2:** Structure of the International Classification of Diseases, 11th Revision (ICD-11) Nutritional Disorders


ICD-11 Nutritional Disorders*
Undernutrition
Undernutrition based on anthropometric and clinical criteria
Undernutrition based on anthropometric and clinical criteria in infants, children and adolescents
Moderate underweight in infants, children and adolescents
Severe underweight in infants, children and adolescents
Moderate wasting in infants, children and adolescents
Severe wasting in infants, children and adolescents
Moderate acute malnutrition in infants, children and adolescents
Severe acute malnutrition in infants, children and adolescents
Moderate stunting in infants, children and adolescents
Severe stunting in infants, children and adolescents
Undernutrition based on anthropometric and clinical criteria in adults
Mild thinness in adults
Moderate thinness in adults
Undernutrition due to specific nutrient deficiencies
Vitamin deficiencies
Vitamin A deficiency
Vitamin A deficiency with night blindness
Vitamin A deficiency with conjunctival xerosis
Vitamin A deficiency with conjunctival xerosis and Bitot’s spots
Vitamin A deficiency with corneal xerosis
Vitamin A deficiency with corneal ulceration or keratomalacia
Vitamin A deficiency with xerophthalmic scars of cornea or blindness
Vitamin D deficiency
Vitamin D deficiency rickets
Vitamin D deficiency osteomalacia
Vitamin E deficiency
Vitamin K deficiency
Deficiencies of B group vitamins
Vitamin B_1_ deficiency
Beriberi
Dry beriberi
Wet beriberi
Wernicke–Korsakoff syndrome
Wernicke encephalopathy
Korsakoff syndrome
Vitamin B_2_ deficiency
Vitamin B_3_ deficiency
Pellagra
Vitamin B_6_ deficiency
Vitamin B_12_ deficiency
Certain specified deficiencies of B group vitamins
Biotin deficiency
Panthotenic acid deficiency
Choline deficiency
Vitamin C deficiency
Scurvy
Scorbutic purpura
Neonatal scurvy
Mineral deficiencies
Iron deficiency
Iron depletion without anaemia
Calcium deficiency
Tetany due to acute calcium deficiency
Neonatal hypocalcaemia
Neonatal osteopenia
Zinc deficiency
Iodine deficiency disorders
Fluorine deficiency
Dental caries due to fluorine deficiency
Magnesium deficiency
Sodium chloride deficiency
Copper deficiency
Selenium deficiency
Keshan disease due to selenium deficiency
Kashin–Beck disease due to selenium deficiency
Chromium deficiency
Manganese deficiency
Molybdenum deficiency
Vanadium deficiency
Certain specified nutritional deficiencies
Essential fatty acid deficiency
Protein deficiency
Overweight, obesity and specific nutrient excesses
Overweight and obesity
Overweight and localized adiposity
Overweight
Overweight in infants, children and adolescents
Risk of overweight in infants and children up to 5 years of age
Overweight in school-aged children and adolescents, 5 to 19 years
Overweight in adults
Localized adiposity
Fat pad
Obesity
Obesity due to energy imbalance
Drug-induced obesity
Obesity hypoventilation syndrome
Leptin-related genetic obesity
Specific nutrient excesses
Vitamin excesses
Hypervitaminosis A
Hypercarotenaemia
Hypervitaminosis D
Megavitamin-B_6_ syndrome
Mineral excesses
Iron overload
Acquired haemochromatosis
Hypercalcaemia
Zinc excess
Sodium chloride excess
Fluorine excess
Aluminium excess
Manganese excess

*Please note the ICD automatically generates residual categories (named ‘other specified…’ or ‘…unspecified’) to include conditions that cannot be assigned to existing entities.

Participants were offered online access to the survey via email. Once the survey was opened, respondents could stop and save answers and continue responding later at their convenience. No hard copies were distributed. The survey was conducted over a period of 34 d between 22 June and 25 July 2015.

Information was collected using twenty-five questions (see online supplementary material) covering the following areas: (i) information about the participant (seven questions); (ii) current use of the ICD (seven questions); and (iii) feedback on the new structure of ICD-11 ND section (eleven questions).

In the first section, participants were asked about their profession and specialization, the type of organization for which they work, whether it is in the private or public sector, and the country where it is located.

The section on current use of the ICD sought to ascertain which version participants are using (ICD-9, ICD-10 or other), how familiar they are with the coding system and how frequently they use the ICD. Participants were asked their opinions about the usefulness of the ICD-9/ICD-10 classification systems as tools for coding nutritional disorders, and the limitations and challenges encountered in using them.

Questions in the third section focused on the new ICD-11 structure of the ND section. Participants were asked their opinion about the level of detail and whether the new ND section covers all nutritional disorders encountered in their work. Additionally, open-ended questions attempted to identify specific challenges or matters of concern in the ICD-11 ND section for coding nutritional disorders.

LimeSurvey^®^, an open-source software tool used by WHO to conduct online surveys, was used to conduct the survey. Summary statistical analyses were performed using the survey tool and Microsoft^®^ Excel.

## Results

**Fig. 1:**
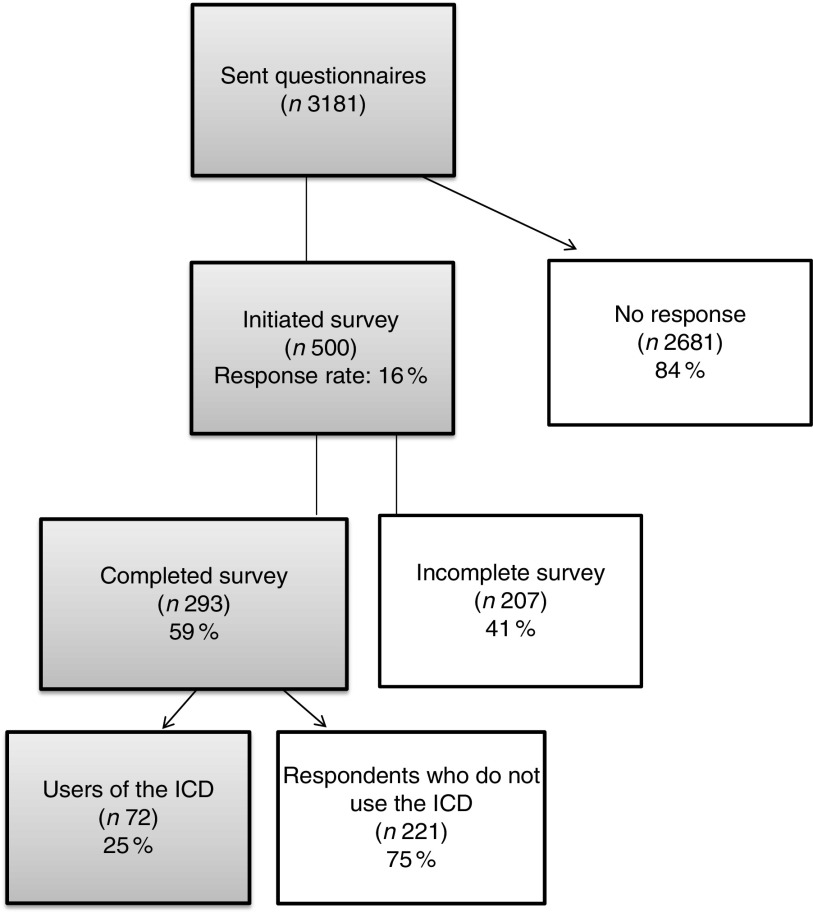
Survey flowchart (ICD, International Classification of Diseases)

Figure 1 presents the survey flowchart. A total of 3181 questionnaires were successfully delivered by email. Of these, 500 participants accessed the survey and 293 submitted a complete questionnaire. Among the 293 participants completing the survey, seventy-two reported using the ICD classification in their current practice while 221 did not. As the survey was designed to obtain feedback from participants familiar with the ICD, results presented below concern the seventy-two ICD users who returned completed questionnaires. Respondents used the ICD mostly for clinical purposes (e.g. many countries require ICD codes to make any drug prescriptions for treatments covered by the public health system), teaching purposes (e.g. use updated disease terms and definitions), financing purposes (e.g. codification of diagnostic and treatment procedures expenditures in the context of hospitalizations) and research projects (e.g. codification of causes of death and morbid conditions).

Survey respondents came from twenty-two countries, with the largest number from the Region of the Americas (31 %) followed by the South-East Asia Region (19 %). Participants from the four remaining WHO regions (African, European, Eastern Mediterranean and Western Pacific) had similar response levels.

The three most common occupations listed by participants were nutritionists (31 %), public health professionals (17 %) and medical doctors (13 %). In medicine, general practice, paediatrics, nutrition and internal medicine were the top four fields of specialization (30 %, 26 %, 13 % and 13 %, respectively). The most common roles included researchers, professors and project coordinators followed by programme leaders, health-care providers/clinicians and senior managers.

The majority of respondents (73 %) used ICD-10 exclusively, 17 % were still using ICD-9 and 10 % reported using both versions. The information obtained on frequency of use showed that almost half of participants used the ICD classification system at least three times per year (46 %), 38 % at least three times per month, and 16 % at least three times per week.

On the usefulness of ICD-9/ICD-10 for coding nutritional disorders, 28 % (*n* 20) ranked them as extremely useful, 26 % (*n* 19) as moderately useful and 31 % (*n* 22) as fairly useful. Eleven respondents (15 %) thought ICD-9/ICD-10 were not useful at all.

Among the limitations participants reported when coding ND with ICD-9 and/or ICD-10, the problems most commonly listed were ‘unclear/confusing grouping’, ‘content not up to date’, ‘missing entities’, ‘unclear, confusing structure’ and ‘entities not consistent’ (Table 3). The main concern expressed by respondents was that ICD-10 was inadequate in terms of covering nutritional condition diagnoses.

**Table 3:** Limitations/challenges identified by stakeholders with the use of ICD-9/ICD-10

	Total number (*n*)	%
Unclear/confusing groupings	28	25·2
Content not up to date	21	18·9
Missing entities	20	18·0
Unclear/confusing structure	17	15·3
Other	13	11·7
Entities not consitent	12	10·8

ICD-9, International Classification of Diseases, 9th Revision; ICD-10, International Classification of Diseases, 10th Revision.

Overall, 25 % of respondents strongly agreed, and 44 % agreed, that the ICD-11 ND section provided a meaningful way to classify nutritional disorders. Only three respondents (4 %) disagreed and nineteen (26 %) were neutral.

To the question ‘Is the level of detail of the new ICD-11 structure for ND appropriate?’, 74 % answered ‘just right’, 8 % ‘too detailed’ and 18 % ‘not enough details’.

**Fig. 2:**
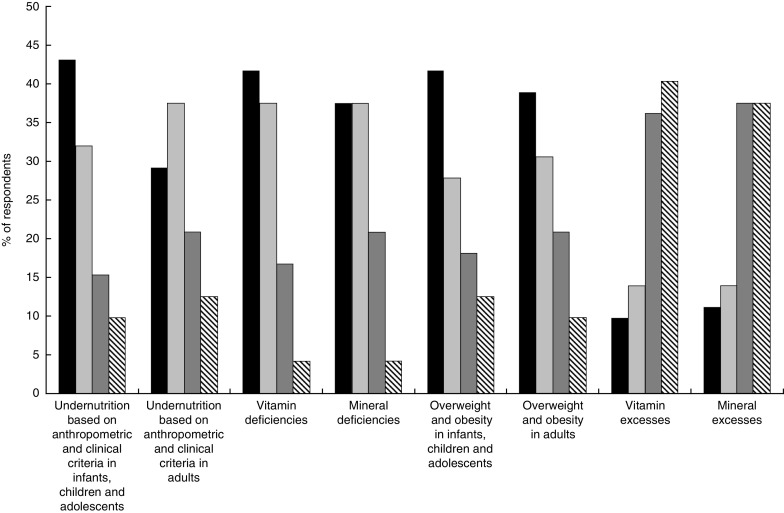
Frequency of use (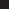, often used (≥3 times/week); 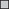, sometimes used (≥3 times/month); 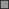, rarely used (≥3 times/year); 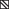, never used) of Nutritional Disorder entities in the new structure of ICD-11 in stakeholders’ day-to-day practice (ICD-11, International Classification of Diseases, 11th Revision)

Figure 2 presents the nutritional conditions in the new structure of ICD-11 most frequently used by respondents. About 40 % of respondents used at least three times per week disorders under the groupings ‘Undernutrition based on anthropometric and clinical criteria in infants, children and adolescents’, ‘Vitamin deficiencies’, ‘Mineral deficiencies’, ‘Overweight and obesity in infants, children and adolescents’ and ‘Overweight and obesity in adults’. About the same proportion of participants reported occasionally using ‘Undernutrition based on anthropometric and clinical criteria in adults’ and ‘Mineral deficiencies’ (at least three times per month).

‘Vitamin excesses’ and ‘mineral excesses’ were the least frequently used groups of nutritional disorders, with 40 and 38 % of participants, respectively, reporting never using them.

Importantly, 78 % (*n* 56) of participants reported that all nutritional disorders were represented in ICD-11 and that their area of specialty was adequately covered. Comments provided by participants for improving the classification included the need for actions to deal with the disease/condition, inclusion of missing disorders (i.e. iodine excess, re-feeding syndrome), more clarity on the transition from previously used terms to new terminology (e.g. kwashiorkor to severe acute malnutrition), recommendations for links to other classifications such as ICF (International Classification of Functioning, Disability and Health), and the need for health-care providers/clinicians/coders to be trained in the use and documentation of the 11th revision once it is released.

Overall, 96 % (*n* 69) of participants reported that the ICD-11 ND section will be a useful improvement over the ICD-10; they expressed appreciation for the new structure, mentioning that it is more comprehensive and specific, includes the main nutritional conditions that are missing in ICD-9 or ICD-10 (e.g. childhood overweight and obesity, stunting, moderate and severe acute malnutrition) and represents an upgrade of the terminologies used. Other positive comments referred to the classification covering population subgroups (i.e. infants, children, adolescents, adults) and displaying information in a clear and precise format.

## Discussion

Feedback from stakeholders around the world suggests that the new structure of the ICD-11 ND section provides a useful improvement over previous versions (ICD-9/ICD-10). It also identifies areas needing improvement before ICD-11 is finalized and adopted. These areas relate mostly to content (adding short and long definition of conditions), adding missing disorders (e.g. iodine excess, re-feeding syndrome), providing more clarity on the transition to new terminology (e.g. kwashiorkor to severe acute malnutrition), recommending links to other classifications of functioning, disability and health, and providing actions to address the disorders.

To the best of our knowledge, this is the first stakeholder survey on the ND section of ICD-11. Other Topic Advisory Groups such as the Quality and Safety TAG have performed similar surveys investigating stakeholders’ views on how to improve the quality and safety applications of ICD-11. Consistent with our results, issues identified by stakeholders when using ICD-9/ICD-10 included missing codes/information/concepts, insufficient updates on current medical knowledge and unclear clustering of categories^(^ ^9^ ^)^.

Concerning mental health, Tyrer *et al*. reported in their study that respondents emphasized that ICD-11 was a more useful tool than ICD-10 in clinical practice when coding personality disorders. Similar to the positive feedback received in our survey to the proposed ND section in ICD-11 (e.g. on the enhanced coverage of population subgroups), improvements mentioned included wider age ranges and an expanded section on pathology^(^ ^10^ ^)^.

Similarly, in a survey conducted by Demoly *et al*., the majority of respondents considered the ICD-10 classification as inappropriate in clinical practice for coding hypersensitivity disorders. The ICD-10 classification was described as unclear, insufficient and inadequate. Missing and inaccurate entities limited coding of allergic diseases^(^ ^11^ ^)^.

Our study has a number of limitations. First, for various reasons (email address could not be found, email system processing problems, recipient’s mailbox was full, problem occurring during delivery, message rejected, permission or security issue), 19 % of email invitations could not be delivered to individual subscribers to the WHO Department of Nutrition for Health and Development’s mailing list, thus excluding them from the survey. Second, sending the questionnaire by email limited the sample to interested parties with access to a computer. Lastly, the survey automatically excluded experts who did not use the classification in their current activities even if, retrospectively, we realize that their feedback might have been useful for the evaluation. It was nevertheless possible to compensate for this last limitation through the public revision process WHO established via the Internet-based ICD-11 platform. All interested parties could participate by submitting proposals for enhancing content and structure^(^ ^8^ ^)^. By the end of April 2016, forty-three proposals had been received for the ND section of Chapter 6 (‘Endocrine, nutritional and metabolic diseases’). Of these, thirty-nine were related to content enhancement (i.e. adding definitions, refining titles, adding/deleting synonyms), one concerned deleting entities (i.e. ‘certain specified deficiencies of B group vitamins’) and three involved hierarchical changes: one to designate the neurological chapter as the primary parent for the ‘Nutritional and toxic disorders of the nervous system’ since all diseases included there are neurological entities; and two to make hierarchical changes in the anthropometric structure (a proposal that had already been captured by the survey being reported in the current paper) and the neonatal hypocalcaemia entity. The public revision process is still ongoing and anyone is welcome to contribute to it.

Notwithstanding the above-mentioned limitations, our results underscore the need for the ICD-11 ND section. Stakeholders expressed appreciation for the new structure. Content enhancements (e.g. considerable expansion of the overweight, obesity and micronutrient excesses categories) are an important step for coding individual patients, collecting and comparing data for global overweight and obesity statistics, and thus for allocating resources and implementing action to address the global burden of overweight, obesity and related health problems.

Similarly, major improvements in content and level of detail of the category ‘undernutrition’ and its sub-categories (e.g. moderate/severe underweight, moderate/severe wasting, moderate/severe stunting, and moderate/severe acute malnutrition in infants, children and adolescents (MAM/SAM)) will permit differentiation between the many forms of undernutrition and will allow correct coding of nutritional disorders in different age groups, which is not possible with ICD-10. Thus, future data collection and monitoring will promote better targeting for interventions aimed at preventing and treating childhood undernutrition. Moreover, the classification’s availability on an electronic platform will greatly facilitate its application.

## Conclusion

There are noticeable differences between ICD-10 and the proposed ICD-11 in the taxonomy of nutritional disorders. The 11th revision is being upgraded to include the full range of nutritional disorders throughout the life cycle, many of which are missing in ICD-10, including undernutrition-related entities based on anthropometric and clinical criteria, as well as overweight and obesity disorders.

Our study documents stakeholders’ overall satisfaction with the comprehensiveness, clarity and coverage of population subgroups of the ND section being proposed for ICD-11. It also identifies areas for improvement before ICD-11 is finalized and adopted in 2018.

The new ND section should be useful to a wide range of health professionals, from nutritionists and researchers to health-care providers and coders. The improved tool is expected to enhance the classification and accurate coding of the full range of nutritional disorders and support clinical care and the attainment of public health objectives for years to come.

## Supplementary material

For supplementary material accompanying this paper visit https://doi.org/10.1017/S1368980016001427. click here to view supplementary material
