# Supplementary material for: Nutritional disorders in the proposed 11th revision of the International Classification of Diseases: feedback from a survey of stakeholders
Source: Public Health Nutr. 2016 Jun 13;19(17):3135–41. doi: 10.1017/S1368980016001427 (PMC5217466; doi:10.1017/S1368980016001427)
Supplement: Supplementary file 1 [file S1368980016001427sup001.docx]

International Classification of Diseases 11^th^ Revision – Nutritional Disorders

**The International Classification of Diseases (ICD) is the global health information standard for mortality and morbidity statistics. It covers all diseases, disorders, injuries and other health related conditions. It is globally used to monitor incidence and prevalence of diseases, to identify health trends and manage resource allocation. Currently, the ICD is undergoing the 11th revision process.**

In order to continue improving ICD’s usability and effective application of the Nutritional Disorders section in the Endocrine, nutritional and metabolic diseases chapter, the Nutrition Department of the WHO is conducting a stakeholders survey. The questions address the following topics:

1. Information about the Participant (7 questions)
2. Current use of International Classification of Diseases (7 questions)
3. Feedback on the new structure of ICD-11 (11 questions)

We highly value your input and would greatly appreciate if you could take some time out of your busy schedule to participate in the survey.

The survey will be open through 25/07/2015. Please participate and help us further improve the ICD-11 Nutritional Disorder Chapter, so that once finalized it will serve you as an effective tool.

Please direct any questions you may have about the survey to [zeitlhuberj@who.int](mailto:zeitlhuberj@who.int)

# Information about the participant

1. **Please select the highest educational degree you have obtained (Please choose all that apply):**
   - Pre-university
   - University degree
   - Non doctoral post graduate degree (e.g. Master’s)
   - Doctoral Degree
   - Other: _____________________________________
2. **Please select the occupation(s) from the list that describe(s) your profession (Please choose all that apply):**
   - Medicine
   - Nursing
   - Midwifery
   - Pharmacy
   - Dietetics
   - Nutrition
   - Health Information Management (coding, medical record administration)
   - Analysis/Statistics
   - Epidemiology
   - Public Health
   - Health Policy
   - Health Care
   - Administration
   - Other:_____________________________________________
3. **What is your field of specialty in medicine? (Only answer this question if the answer/one of the answers in Q2 was “medicine”)**
   - Cardiology
   - Endocrinology
   - Internal Medicine
   - Gastroenterology
   - General Practice
   - Nephrology
   - Neurology
   - Nuclear Medicine
   - Neonatology
   - Paediatrics
   - Other: _____________________________________________
4. **Please select the type of organization(s) you work for** (Please choose **all** that apply):
   - Self-employed
   - University, academic health science center or research institute
   - International Organization
   - Insurance company, program or fund
   - Hospital/Clinic
   - Other:________________________________________________
5. **Do you work in the private or public sector? (**Please choose **only one** of the following)
   - Private
   - Public
   - Both
6. **Please select the role(s) you have in the organization** (Please choose **all** that apply):
   - Researcher
   - Health Care Provider/Clinician
   - Program Leader (e.g. manager)
   - Project coordinator
   - Senior Management (e.g. CEO, Director)
   - Health record coder
   - Analyst/Statistician
   - Administrator
   - Professor
   - Student
   - Other:___________________________________________
7. **Which country are you currently located in?**

____________________________________________________

# Current use of international classification of diseaes

1. **In your current practice, do you use the ICD classification system? (Please choose only one of the following)**
   - Yes¨
   - No

## CONTINUE answerng the survey if the answer in **QUestion 8** was **“yes”.** if the answer was “**No**”, please go to page 10.

1. **Which version of the ICD do you use?** (Please choose all that apply)
   - ICD-9
   - ICD-10
   - Other: ______________________________________
2. **How familiar are you with the ICD coding system?** (Please choose the appropriate response to each item)

|  | **Very**  **familiar** | **Fairly familiar** | **Moderately** | **Fairly unfamiliar** | **Completely unfamiliar** |
| --- | --- | --- | --- | --- | --- |
| **ICD-9** |  |  |  |  |  |
| **ICD-10** |  |  |  |  |  |

1. **As part of your work, how often do you use the ICD classification system?** (Please choose **only one** of the following)
   - Often (at least 3 times a week)
   - Sometimes (at least 3 times a month)
   - Rarely (at least 3 times a year)
   - Never
2. **How useful have the ICD-9/ICD-10 classification systems been as tools for coding nutritional disorders in your practice?** (Please choose **only one** of the following)
   - extremely useful
   - fairly useful
   - moderately useful
   - not useful at all
3. **Please, identify main limitations/challenges you found when using ICD-9 and/or ICD-10 for the purpose of classifying nutritional disorders** (Please choose **all** that apply):
   - missing entities
   - unclear/confusing groupings
   - unclear/confusing structure
   - content not up-to-date
   - entities not consistent
   - other
4. **Please provide further information (if the answer to Question 13 is “other”)**

|  |
| --- |

# feedback on the new structure OF icd-11 nutritional disorders

| **To answer the following questions, please open the overview of the old and the new structure of the Nutritional Disorders (ND) Chapter by clicking the following links:** [ICD-11 ND structure](ICD%20structure%20PDFs%20and%20word%20docs/ICD-11%20New%20Structure.pdf) **& ICD-10 ND structure.**  **If you would like to see the entire ICD 11th Revision Beta Browser online, you can register** [here.](http://apps.who.int/classifications/icd11/browse/l-m/en) |
| --- |

1. **Do you agree that the ICD-11 ND Chapter provides a meaningful way to classify Nutritional Disorders?** (Please choose **only one** of the following)
   - strongly agree
   - agree
   - neutral
   - disagree
   - strongly disagree
2. **Is the level of detail of the new ICD-11 structure for ND appropriate?** (Please choose **only one** of the following)
   - too detailed
   - just right
   - not enough details
3. **Please provide further information: (if the answer to Question 16 is “not enough details”)**

|  |
| --- |

1. **Are the nutritional disorders you encounter in your work represented in the new ICD-11?** (Please choose **only one** of the following)
   - Yes
   - No
2. **Please provide further information: (if the answer to Question 18 is “No”)**

|  |
| --- |

1. **Do you think your area of specialty is adequately covered in the ICD-11 ND chapter?** (Please choose **only one** of the following)
   - Yes
   - No
2. **Please provide further information: (if the answer to Question 20 is “No”)**

|  |
| --- |

1. **Do you think that the ICD-11 ND chapter will be a useful improvement over ICD-10?** (Please choose **only one** of the following)
   - Yes
   - No
2. **Please provide further information: (if the answer to Question 22 is “No”)**

|  |
| --- |

1. **Of the ICD-11 ND entities listed, which are the most frequently encountered in your day-to-day practice?** (Please choose the appropriate response for each item)

|  | **often used** (at least 3 times a week) | **sometimes used** (at least 3 times a month) | **rarely used** (at least 3 times a year) | **never used** |
| --- | --- | --- | --- | --- |
| **Undernutrition based on anthropometric and clinical criteria in infants, children and adolescents** |  |  |  |  |
| **Undernutrition based on anthropometric and clinical criteria in adults** |  |  |  |  |
| **Vitamin Deficiencies** |  |  |  |  |
| **Mineral Deficiencies** |  |  |  |  |
| **Overweight and obesity in infants, children and adolescents** |  |  |  |  |
| **Overweight and obesity in adults** |  |  |  |  |
| **Vitamin Excesses** |  |  |  |  |
| **Mineral Excesses** |  |  |  |  |

1. **Please add any further comments or matters of concern you might have on the International Classification of Diseases 11^th^ Revision – Nutritional Disorders:**

|  |
| --- |

**Thank you for participating in the survey!**

**Department of Nutrition for Health and Development (NHD)**

**World Health Organization, Geneva**
